# Vancomycin area under the curve-guided monitoring in pediatric patients

**DOI:** 10.5935/0103-507X.20220009-en

**Published:** 2022

**Authors:** Ronaldo Morales Junior, Vanessa D’Amaro Juodinis, Isabela Cristina Pinheiro de Freitas Santos, Camila Canuto Campioni, Flávia Gatto de Almeida Wirth, Livia Maria Goncalves Barbosa, Daniela Carla de Souza, Silvia Regina Cavani Jorge Santos

**Affiliations:** 1 Department of Pharmacy, Hospital Sírio- Libanês - São Paulo (SP), Brazil.; 2 Pediatric Intensive Care Unit, Hospital Sírio- Libanês - São Paulo (SP), Brazil.; 3 Center of Clinical Pharmacokinetics, Faculdade de Ciências Farmacêuticas, Universidade de São Paulo - São Paulo (SP), Brazil.

**Keywords:** Vancomycin, Drug monitoring, Pharmacokinetics, Child

## Abstract

**Objective:**

To assess the percentage of vancomycin area under the curve/minimum inhibitory concentration target attainment in pediatric patients after the empirical dose regimen and to demonstrate the applicability of this method for vancomycin monitoring.

**Methods:**

A retrospective cohort study was performed including pediatric patients with normal renal function admitted between January 2020 and December 2020. The one-compartment model with first-order kinetics was used to estimate the pharmacokinetic parameters, and the area under the curve was calculated by the trapezoidal rule. The therapeutic target was defined as area under the curve/minimum inhibitory concentration ≥ 400 and < 600. The Chi-squared test was applied to compare the percentage of target attainment over age groups, while the pharmacokinetic parameters were compared by the Kruskal-Wallis test with Dunn’s test for post hoc analyses. We considered significant p-values < 0.05.

**Results:**

In total, 42 pairs of vancomycin levels were analyzed from 17 patients enrolled in this study. After empirical vancomycin daily dosing, the therapeutic target was achieved in five (29%) patients; four patients (24%) had a supratherapeutic initial area under the curve/minimum inhibitory concentration value (> 600mg.h/L), and eight (47%) patients had subtherapeutic values (< 400mg.h/L). The most identified pathogens were *Staphylococcus spp.* (n = 7). Trough levels and areas under the curve showed moderate correlation values (R^2^ = 0.73). Acute kidney injury occurred in one (6%) patient.

**Conclusion:**

Most patients did not reach the therapeutic target with a vancomycin empirical dose regimen, and the implementation of area under the curve-based dosing using two sample measurements allowed for real-time dose adjustments based on individuals’ pharmacokinetic parameters.

## INTRODUCTION

Vancomycin is a glycopeptide antibiotic commonly prescribed to treat invasive *Gram*-positive bacterial infections, including methicillin-resistant *Staphylococcus aureus* (MRSA) and ampicillin-resistant Enterococci.^(1)^ The best clinical and bacteriological outcomes with vancomycin use were found when the 24-hour area under the serum concentration-time curve (AUC) reached a value of 400 times the minimum inhibitory concentration (MIC) of the microorganism, that is, AUC_24_/MIC > 400.^(2,3)^

The first vancomycin monitoring guideline, published in January 2009 as a joint effort by the Society of Infectious Disease Pharmacists (SIDP), American Society of Hospital Pharmacists (ASHP) and Society of Infectious Diseases of America (IDSA), recommended trough level vancomycin monitoring in adult patients and suggested minimum serum levels between 15 and 20mg/L as sufficient to achieve AUC_24_/MIC > 400.^(4)^ Pediatric patients were excluded from these monitoring recommendations.

Several studies have been performed attempting to identify ideal trough levels to be used as surrogate markers of AUC_24_/MIC in children and newborns. However, widespread variability of pharmacokinetic parameters was found in these patients, corroborating the need for AUC-guided monitoring to assess cumulative drug exposure over a 24-hour period.^(5-9)^

A newly revised consensus guideline for vancomycin dosing and monitoring was published in 2020 with the collaboration of the Pediatric Infectious Disease Society (PIDS). The update recommended that trough levels should not be used as predictors of vancomycin AUC.^(10)^ The new guideline recommends AUC-based monitoring according to pharmacokinetic and pharmacodynamic (PK/PD) concepts in both adult and pediatric patients to maximize antibiotic efficacy while minimizing nephrotoxicity risk.

The vancomycin AUC can be estimated using two steady-state vancomycin levels drawn during the same interval and calculating the area by the trapezoidal rule.^(11)^ Alternatively, Bayesian software can be used, but its high cost is the main limitation of this approach.^(12)^

Therefore, following the new recommendations and given the familiarity of our pharmacists with pharmacokinetic calculations, we transitioned from trough-only monitoring to a two-sample AUC estimation in the pediatric units of our hospital. The purpose of this study was to assess the percentage of vancomycin AUC/MIC target attainment in pediatric patients after the empirical dose regimen and to demonstrate the applicability of this method for vancomycin monitoring.

## METHODS

### Patient population and study design

This study was a retrospective cohort study performed in the pediatric intensive care unit (ICU) of *Hospital Sírio-Libanês*, a philanthropic hospital complex in São Paulo, Brazil. Pediatric patients (aged < 18 years old) admitted between January 2020 and December 2020 who received vancomycin for at least 48 hours were included in the study. Patients who had acute or chronic renal failure with an estimated creatinine clearance less than 60mL/min/1.73m^2^ using the modified Schwartz equation^(13)^ or receiving renal replacement therapy were excluded. Demographic, clinical, and microbiological data were collected. The study protocol was approved by the local Ethics Committee and registered under number CAAE 41216320.0.0000.5461.

Information was extracted from the electronic medical records. Data variables included age, sex, total body weight (TBW), height (HT), serum creatinine (Scr), indication for vancomycin, vancomycin serum levels, microbiology cultures (blood, urine, catheter tip, tracheal aspirate, wound and cerebrospinal fluid), vancomycin dose, date and time of drug administration and blood sampling.

### Blood sampling and laboratory assay

Vancomycin empirical therapy started at 10 to 15mg/kg four times per day, with a one-hour pump infusion. Two blood samples were collected at the second and sixth hours after starting the infusion after steady-state achievement (after four doses had been administered or at 24 hours; the time point was approximately equal to five times the drug half-life). If necessary, doses were adjusted, and the serum levels were rechecked to ensure target achievement until the patients were discharged or transferred to another hospital.

Serum vancomycin concentrations were measured by fluorescence immunoassay (VANC3, Cobas, Roche Diagnostics, Indianapolis, United States). The limit of detection of this assay was 4µg/mL.

### Pharmacokinetic/pharmacodynamic analysis

The implementation of the AUC-based vancomycin monitoring protocol occurred over a period of one year, from January to December 2020. We built a dose calculator using Microsoft Excel software, based (Appendix 1) on the one-compartment model. The users enter patient information, dosing regimen data and date/time/value of paired levels drawn, and the spread sheet calculates the AUC and pharmacokinetic values. Users can input different dosing regimens and MIC targets to verify predicted AUC/MIC values based on individual pharmacokinetic parameters. First-order kinetics equations were used to estimate the predicted peak and trough, elimination rate constant (K_el_), biological half-life (T_1/2_), vancomycin clearance (CLvan) and volume of distribution (V_d_). The vancomycin AUC of dose intervals (AUC_tau_) was estimated by the logarithmic trapezoidal rule, in which tau is 6 hours for drugs administered every 6 hours and then multiplied by four to calculate the 24-hour AUC.^(14)^ The equations used in the Microsoft Excel spreadsheet were validated by Pai et al., who demonstrated that these pharmacokinetic parameters and daily AUC could be determined with reasonable precision and low bias using near steady-state, postdistributional peaks (1 to 2 hours after the end of infusion) and trough concentrations within the same dosing interval.^(14,15)^ Therapeutic targets were defined as AUC^ss^_0-24_/MIC ≥ 400 and < 600. When it was not possible to identify the pathogen, the vancomycin MIC was assumed to be 1mg/L following the 2020 guideline recommendations.^(10)^

### Study outcomes and definitions

The primary outcome was the percentage of target attainment (PTA) after empirical dosing. Secondary outcomes included the rate of vancomycin-associated acute kidney injury (AKI) and the relationship between the estimated AUC and trough values.

Vancomycin-associated nephrotoxicity was defined as an increase in Scr of 0.5mg/dL or 50% from baseline and was more highly attributable to vancomycin than to another cause by the primary team.

### Statistical analysis

Microsoft Excel software, version 2007 (Microsoft Corporation, Redmond, WA, USA), and GraphPad Prism software, version 8.0 (GraphPad Software, Inc., San Diego, CA, USA), were used to organize and present population data and to perform correlation analysis. The data related to demographic characteristics of the patient population, as well as the results related to doses, serum levels and pharmacokinetic parameters, are expressed as medians (interquartile range - IQR) because nonparametric statistics were applied, and a small number of outliers were observed. To assess the correlation between the calculated AUC_24_ and trough vancomycin serum concentrations, Pearson’s linear correlation analysis was performed. For pharmacokinetic parameter evaluations in this study, patients aged < 1 year old, patients aged ≥ 1 old and < 7 years old, and patients aged ≥ 7 years old were allocated to Group 1 (G1), Group 2 (G2) and Group 3 (G3), respectively, given that vancomycin parameters differ by age.^(16)^ The Chi-squared test was applied to compare the PTA among the groups. The pharmacokinetic parameters were compared by the nonparametric Kruskal-Wallis test with Dunn’s test for *post hoc* analyses. We considered significant p-values < 0.05.

## RESULTS

A total of 19 subjects were screened. Two patients were excluded because they were on renal replacement therapy. The remaining 17 patients met the inclusion criteria for this investigation, seven female (41%) and ten male patients (59%) and accounted for 42 pairs of vancomycin levels for therapeutic drug monitoring. The patients’ demographic and clinical characteristics are shown in table 1. Since our hospital is a referral center for pediatric liver transplantation, most of the patients were hospitalized for liver transplantation (n = 8; 47%) and other abdominal surgeries (n = 3; 18%), followed by respiratory complications (n = 2; 12%) and meningitis (n = 2; 12%). The most identified pathogens were *Staphylococcus spp.,* isolated in tracheal aspirate (58%), blood (14%), catheter tips (14%) and cerebrospinal fluid (14%).

**Table 1:** Demographic and clinical information of pediatric patients

Characteristic	Value
Age (years)	1.5 (0.8 - 5.0)
< 1	5 (29)
≥ 1 and < 7	8 (47)
≥ 7	4 (24)
Male sex	10 (59)
Body weight (kg)	9.7 (7.7 - 22.9)
Height (cm)	79 (66 - 110)
Creatinine clearance (60mL/minute/1.73m^2^)	151.9 (132.6 - 159.2)
Length of stay (days)	14 (6 - 47)
Initial vancomycin dose (mg/kg)	11.1 (9.8 - 12.5)
Indication for vancomycin therapy	
Abdominal/pelvic	10 (59)
Pulmonary	3 (18)
Bacteremia	2 (12)
Central nervous system	2 (12)
*Gram-*positive bacteria isolated	
*Staphylococcus aureus*	5 (29)
*Staphylococcus epidermidis*	2 (12)
*Enterococcus faecium*	1 (6)
*Viridans group streptococci*	1 (6)
*Streptococcus pneumoniae*	1 (6)

Values are expressed as median (interquartile range) or n (%).

**Figure 1:**
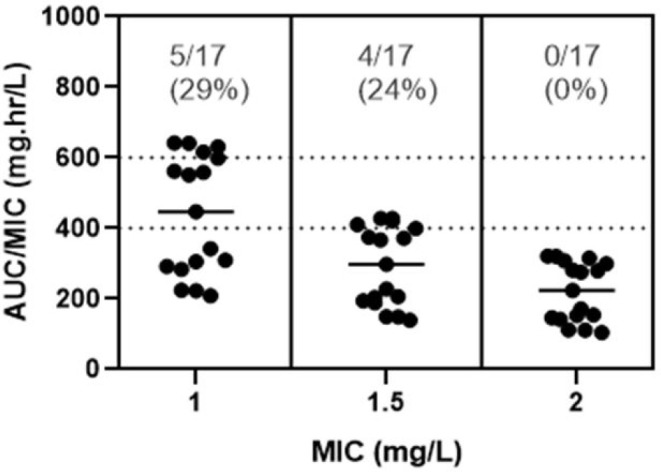
Overall percentage of target attainment after vancomycin empirical dose regimen. AUC - area under the curve; MIC - minimum inhibitory concentration.

After empirical vancomycin daily dosing, the therapeutic target against *Gram-*positive bacteria with a MIC of 1mg/L was achieved in five of 17 (29%) patients. There was no statistically significant difference in PTA between the groups (p = 0.355), and there was also no direct correlation between dose variations between 10 and 15 mg/kg q6h and AUC (p = 0.0586). Only four patients (24%) had a supratherapeutic initial AUC/MIC value (> 600mg.h/L) compared with eight (47%) with a subtherapeutic value (< 400mg.h/L). In those who did not reach the therapeutic target with the empirical regimen, we were able to perform at least one more AUC calculation after the dose adjustment in seven patients based on their individual pharmacokinetic parameters, and then six of them reached the target. The PTA of the empirical dose regimen for different MIC values is shown in figure 1, and the population data of the calculated pharmacokinetic parameters are shown in table 2.

**Table 2:** Estimated pharmacokinetic after vancomycin empirical regimen by group

Kinetic parameter	G1 (< 1 year) n = 5	G2 (≥ 1 and < 7 years) n = 8	G3 (> 7 years) n = 4	p value
*k*_e_ (hour^-1^)	-0.23 (-0.24 --0.22)	-0.25 (-0.33 --0.24) *	-0.18 (-0.19 --0.16)	0.042
Peak (mg/L)	17.9 (14.4 - 34.1)	35.3 (19.9 - 40.3)	26.8 (15.6 - 37.3)	0.173
Trough (mg/L)	7.9 (4.3 - 11.2)	7.5 (6.0 - 9.2)	10.6 (7.3 - 14.0)	0.416
*T*_1/2_ (hours)	3.1 (2.9 - 3.2)	2.7 (2.1 - 3.0) †	3.8 (3.6 - 4.7)	0.034
CL_van_ (mL/minute/kg)	2.1 (1.7 - 3.2)	1.6 (1.1 - 2.3)	2.0 (1.1 - 3.0)	0.574
*V*_d_ (L/kg)	0.7 (0.5 - 0.8)	0.3 (0.2 - 0.7)	0.6 (0.3 - 1.2)	0.317
AUC_tau_ (mg.h/L)	77.4 (56.1 - 137.7)	125.7 (83.2 - 151.8)	113.6 (68.8 - 155.6)	0.444
AUC_24_ (mg.h/L)	309.8 (224.3 - 551.0)	502.8 (332.8 - 607.2)	454.2 (275.1 - 622.3)	0.444

k_e_ - elimination rate constant; *T*_1/2_ - biological half-life; CL_van_ - vancomycin clearance; V_d_ - volume of distribution; AUC_tau_ - area under the curve-time curve of dosing interval; AUC_24_ - 24-hour area under the curve-time curve.

* p = 0.045 compared to Group 3; † p = 0.037 compared to Group 3.

**Figure 2:**
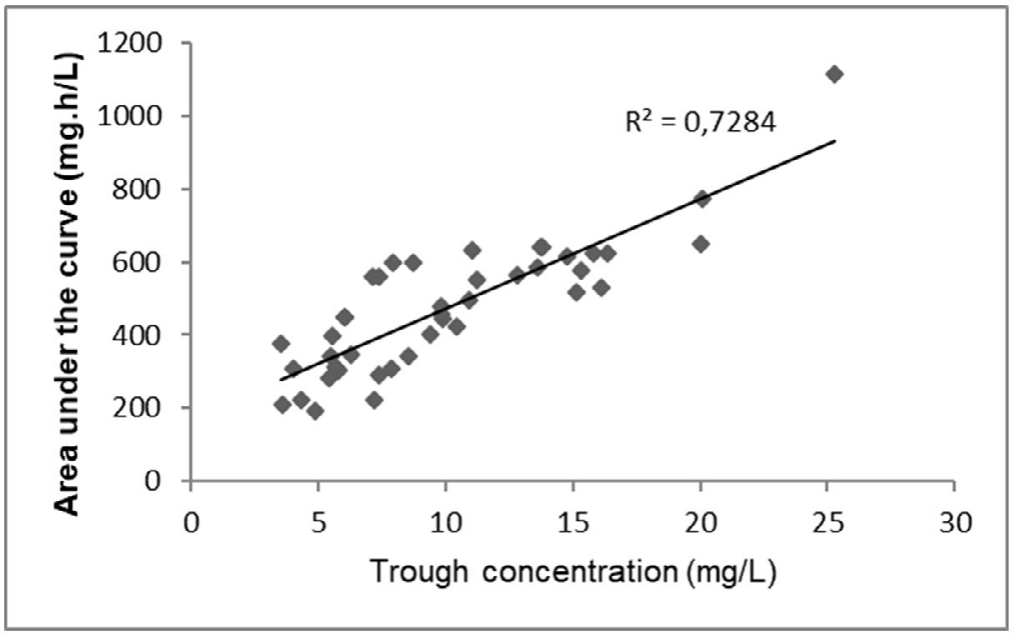
Scatter plot of observed vancomycin trough concentrations and the calculated 24-hour area under the vancomycin concentration-time curve at steady state.

Considering all 42 AUC calculations performed, the relationship between the vancomycin trough levels and the calculated AUC is represented in figure 2. When the observed AUC values were between 400 and 600mg.h/L (n = 18), the median trough was 9.9 (8.1 - 12.4) mg/L. Additionally, ten (56%) of the 18 corresponding troughs were less than 10mg/L, 15 (83%) were less than 15mg/L, and eight (44%) were between 10 and 20mg/L. Additionally, in six cases, the trough levels were less than 20mg/L but nevertheless presented AUC values greater than 600mg.h/L. All medical prescriptions were fully evaluated, and no clinically relevant drug interactions that could affect vancomycin serum levels were identified.

Vancomycin-related nephrotoxicity occurred in one (6%) patient. Regarding the four patients who had an AUC value of 600mg.h/L or greater, the daily dose was immediately reduced, and nephrotoxicity was not experienced in any of them during hospitalization. One patient developed red man syndrome after a 1-hour infusion; then, a longer infusion duration was required. No deaths were registered during the period of study.

Inappropriate vancomycin concentration laboratory draws (i.e., due to incorrect timing or those missed completely) occurred in one patient (6%) after the AUC-based monitoring implementation.

## DISCUSSION

In the present study, only 29% of patients reached the therapeutic target with a vancomycin empirical dose regimen. Most patients had subtherapeutic levels (47%), and we had to increase their daily doses. These findings could not be explained by the initial diagnoses, infectious focuses or possible drug interactions. The need for increasing daily doses to reach adequate AUC/MIC targets has been documented in critically ill pediatric patients. Le et al. noted that daily doses of 60 to 70mg/kg were required to achieve the target AUC/MIC in 75% of pediatric patients, and Pires et al. recommended a minimum empirical dose of 60mg/kg per day to achieve the desired outcome.^(17,18)^

Before the 2020 guideline, the reference trough range (10 - 20mg/L) was proposed to guarantee PK/PD target achievement.^(4)^ However, we found that the AUC target was achieved even with trough values less than 10mg/L in 56% of cases, and AUCs greater than 600mg.h/L were obtained with trough values less than 20mg/L in 14% of cases, suggesting that drug serum monitoring by trough levels might lead to excessive dosing. Chhim et al. evaluated vancomycin dosing practices in pediatric patients and found that trough concentrations did not correlate well with the predicted AUC in children receiving the recommended vancomycin daily dose.^(19)^ Therefore, in accordance with the 2020 guidelines, AUC direct calculation might be a more reasonable monitoring approach to ensure antimicrobial coverage.

Studies evaluating vancomycin-related AKI in pediatric patients have been limited, with an incidence rate ranging from 8.8% to 19%.^(20-24)^ Le et al. reported that a vancomycin AUC of ≥ 800mg.h/L and trough concentrations of ≥ 15mg/L were associated with a greater than 2.5-fold increased risk of nephrotoxicity, and they found a statistically significant correlation between vancomycin levels and AKI.^(21)^ Consequently, the 2020 guideline reinforced the AUC estimation as a plausible method for reducing the risk of AKI. We decided to retain the target up to 600mg.h/L, and we had a low rate of vancomycin-related AKI (6%).

There are several limitations regarding this study. First, this study was a single-center experience with a small sample size, and we did not have a comparator group, so caution is necessary when generalizing our results. Second, we used a calculated creatinine clearance determined by Schwartz’s method as a renal function measurement, instead of a 24-hour creatinine measurement. Third, we used the MIC value from surveillance databases instead of clinical laboratory data when it was not possible to identify the pathogen. Finally, it was not the purpose of this study to assess clinical outcomes or compare treatment efficacy among those who reached or did not reach the therapeutic target, and in some cases, we were not able to follow patients’ entire clinical courses because they were transferred to another hospital. Further research is needed to investigate the correlation of AUC target attainment with clinical outcomes in this population.

Nevertheless, this study showed that implementation of AUC-based dosing using two sample measurements was feasible for vancomycin serum level monitoring in pediatric patients. In addition, we found great variability in pharmacokinetic parameters between the patients, and the use of individual pharmacokinetic profiles allowed for specific dose adjustments to improve the target attainment against *Gram-*positive strains with an MIC of 1mg/L.

## CONCLUSION

Most of the patients did not reach the therapeutic targets against *Gram-*positive bacteria with a minimum inhibitory concentration of 1mg/L after the vancomycin empirical dose regimen. With an area under the curve-guided monitoring strategy, it was possible to estimate the pharmacokinetic parameters and individualize the therapy in real time to improve target attainment.

While trough-based monitoring might overexpose patients to vancomycin, area under the curve calculations plus pharmacokinetic and pharmacodynamic correlations can be successfully implemented, constituting a feasible alternative to Bayesian-controlled software for institutions that cannot afford it.
